# Cost-utility analysis of curative and maintenance repetitive transcranial magnetic stimulation (rTMS) for treatment-resistant unipolar depression: a randomized controlled trial protocol

**DOI:** 10.1186/s13063-020-04255-9

**Published:** 2020-04-05

**Authors:** Samuel Bulteau, Andrew Laurin, Christelle Volteau, Cécile Dert, Lydie Lagalice, Solène Schirr-Bonnans, Nicolas Bukowski, Marie Guitteny, Luc Simons, Clémence Cabelguen, Anne Pichot, Fabienne Tessier, Annabelle Bonnin, Adeline Lepage, ACOUSTIM Investigators Group, Jérôme Attal, Jean-Marie Batail, René Benadhira, Djamila Bennabi, Caroline Berjamin, Maxime Bonnin, Noomane Bouaziz, Jérôme Brunelin, Maxime Bubrovszky, Benjamin Calvet, Irina-Alexandra Catana, Macarena Cuenca, Gaelle Dall’ Igna, Dominique Drapier, Laurine Egreteau, Wissam El-Hage, Filipe Galvao, Ghina Germaneau, Bénédicte Gohier, Emmanuel Haffen, Ghassan Harika, Guillaume Ifrah, Némat Jaafari, Isabelle Jalenques, Dominique Januel, Redwan Maatoug, Bruno Millet, Clément Nathou, Benjamin Petit, Damien Pierre, Marion Plaze, Emmanuel Poulet, Gabriel Robert, Maud Rotharmel, Marine Rozet, David Szekely, Simon Taib, Benoît Trojak, Antoine Yrondi, HUGOPSY Network, Jean-Marie Vanelle, Anne Sauvaget, Valery-Pierre Riche

**Affiliations:** 1 Centre Hospitalier Universitaire de Nantes, F-44000 Nantes, France; 2 Réseau HUGOPSY, Université de Rennes, Rennes, France

**Keywords:** Repetitive transcranial magnetic stimulation (rTMS), Long-term, Maintenance, Treatment-resistant depression, Cost-effectiveness, Cost-utility, Prognosis, Major depressive disorder, Health-economic

## Abstract

**Background:**

Depression is a debilitating and costly disease for our society, especially in the case of treatment-resistant depression (TRD). Repetitive transcranial magnetic stimulation (rTMS) is an effective adjuvant therapy in treatment-resistant unipolar and non-psychotic depression. It can be applied according to two therapeutic strategies after an initial rTMS cure: a further rTMS cure can be performed at the first sign of relapse or recurrence, or systematic maintenance rTMS (M-rTMS) can be proposed. TMS adjuvant to treatment as usual (TAU) could improve long-term prognosis. However, no controlled study has yet compared the cost-effectiveness of these two additional rTMS therapeutic strategies versus TAU alone.

**Methods/design:**

This paper focuses on the design of a health-economic, prospective, randomized, double-blind, multicenter study with three parallel arms carried out in France. This study assesses the cost-effectiveness of the adjunctive and maintenance low frequency rTMS on the right dorsolateral prefrontal cortex versus TAU alone. A total of 318 patients suffering from a current TRD will be enrolled. The primary endpoint is to investigate the incremental cost-effectiveness ratio (ICER) (ratio costs / quality-adjusted life-years [QALY] measured by the Euroqol Five Dimension Questionnaire) over 12 months in a population of patients assigned to one of three arms: systematic M-rTMS for responders (arm A); additional new rTMS cure in case of mood deterioration among responders (arm B); and a placebo arm (arm C) in which responders are allocated in two subgroups: sham systematic M-rTMS and supplementary rTMS course in case of mood deterioration. ICER and QALYs will be compared between arm A or B versus arm C. The secondary endpoints in each three arms will be: ICER at 24 months; the cost-utility ratio analysis at 12 and 24 months; 5-year budget impact analysis; and prognosis factors of rTMS. The following criteria will be compared between arm A or B and arm C: rates of responders; remission and disease-free survival; clinical evolution; tolerance; observance; treatment modifications; hospitalization; suicide attempts; work stoppage; marital / professional statues; and quality of life at 12 and 24 months.

**Discussion:**

The purpose of our study is to check the cost-effectiveness of rTMS and we will discuss its economic impact over time. In the case of significant decrease in the depression costs and expenditures associated with a good long-term prognosis (sustained response and remission) and tolerance, rTMS could be considered as an efficient treatment within the armamentarium for resistant unipolar depression.

**Trial registration:**

ClinicalTrials.gov, NCT03701724. Registered on 10 October 2018. Protocol Amendment Version 2.0 accepted on 29 June 2019.

## Background

Depression is a debilitating difficult-to-treat disease associated with enormous costs for our society. According to the Global Burden Study in 2010, it is a leading cause of morbidity and disability worldwide, thus a public-health priority calling for cost-effective interventions [1]. Affecting the whole lives 24% of French people [2], the direct and indirect yearly cost for depression is estimated at 14 billion Euros in France [3]. These expenditures include co-morbidities such as substance abuse, suicide attempts and suicide achieved, somatic multi-morbidities, loss of production, working stoppages, the increased use of the healthcare system (hospitalization, etc.), the impact on the quality of life and its socio-professional aftermath [3–5]. Despite appropriate treatment, 40% of patients do not respond to initial antidepressant treatment and 20% present with a chronic form of depression (> 2 years despite standard treatment administered correctly) [6]. Typically, treatment-resistant depression (TRD) refers to an absence of remission after at least two different antidepressant lines at an effective dosage over a period of 6 weeks during the current episode. Indeed, 30%–40% of patients with major depression hold disabling symptoms after several antidepressant treatments [7–9]. Even in the cases of initial remission, the study of reference for pharmacotherapy, STAR*D, shows a 43% rate of relapse at 1 year [7, 10]. The economic cost of depression is much higher in the case of TRD [11, 12] and is associated to the higher rate of medication non-observance at around 63% [5]. Depression after two antidepressant agents increases the cost of care by 40%–100% in comparison with non-TRD [13]. Moreover, in 2000, a meta-analysis of Dimatteo et al. [14] demonstrated that the non-observance rate of non-psychiatric treatments is three time higher among patients with anxio-depressive syndrome increasing its global burden. Care for depression is therefore a challenge with important direct and indirect costs in the short and long term, especially in its treatment-resistant form.

The gold-standard treatment of TRD is still electroconvulsive therapy (ECT), especially for depression with psychotic symptoms, severe resistance to pharmacological therapies, or life-threatening episodes, with a 48% response rate in ultra-resistant episodes [5, 15]. Repetitive transcranial magnetic stimulation (rTMS) is a non-invasive, focal and cortical stimulation technique enhancing neuronal plasticity throughout the modulation of cortical excitability [16]. This technique is an interesting alternative to ECT for resistant and middle to severe unipolar depression without psychotic symptoms [17], especially in conjunction with antidepressant chemotherapy [18, 19]. Two types of stimulations are widely applied – high frequency on the left dorsolateral prefrontal cortex (DLPFC) and low frequency on the right DLPFC – with evidence suggesting similar efficacy [20], and with the advantage for the 1 Hz protocol to be faster and better tolerated [21]. Since 2008, high frequency rTMS on the left DLPFC is validated in the United States by the Food and Drug Administration (FDA) to treat unipolar depression after the failure of one antidepressant treatment based on the study by O’Rearden et al. [22] involving 301 participants. Similar efficacy was shown among 170 patients with unipolar depression in France using low frequency rTMS on the right DLPFC [23]. In international guidelines, rTMS in the acute phase reached the best level of evidence (level 1) [17] after failure of one antidepressant. rTMS has the advantage of being somatic and cognitively well tolerated [24], without drug interaction [25], and improving the quality of life [26] with a huge rate of observance (an average rate of 3% for dropouts) [21].

However, the rates of relapse and recurrence at 6-month follow-up may be comparable to the ECT [27]. These rates are in the range of 60% without consolidation to 30% with consolidation (pharmacological therapies or consolidation ECT) [28]. Having these comments, two therapeutic strategies are possible: a further rTMS cure can be performed at the first sign of relapse or recurrence [29], especially for patients with a good previous response [30]; or systematic maintenance rTMS (M-rTMS) can be carried out in the immediate future [31]. It is, therefore, essential to evaluate the cost-effectiveness of these alternatives treatment for TRD. It is already established that rTMS is more cost-effective (efficiency) than placebo [32] and rTMS is more efficient than a third-line antidepressant treatment in an Australian context [33]. But no controlled study compared in the long term (12 months) the cost-effectiveness of these two rTMS therapeutic strategies versus treatment as usual (TAU) alone (any therapeutic strategy agreed to by patients and psychiatrists including medication, ECT, and psychotherapy). Our main hypothesis is that additional M-rTMS will be more cost-effective at 1 year than TAU alone.

### Specific aims

The aim of this health-economic study is to investigate the incremental cost-effectiveness ratio (ICER) (ratio costs / quality-adjusted life-years [QALY]) of the curative and maintenance rTMS versus TAU over 12 months in a population of patients suffering from unipolar TRD (Montgomery and Asberg Rating Scale [MADRS] score of ≥ 20). QALY evaluation is measured by the Euroqol Five Dimension Questionnaire (EQ-5D). Moreover, this trial evaluates the budget impact of the most efficient strategy at 5 years, prognosis factors of successful maintenance after rTMS response to a cure, the cost–utility ratio (costs / QALY), and the cost-effectiveness analysis (life years gains [LYG]) (costs/LYG) at 24 months.

## Methods/design

### Study design, setting, and recruitment

**Fig. 1:**
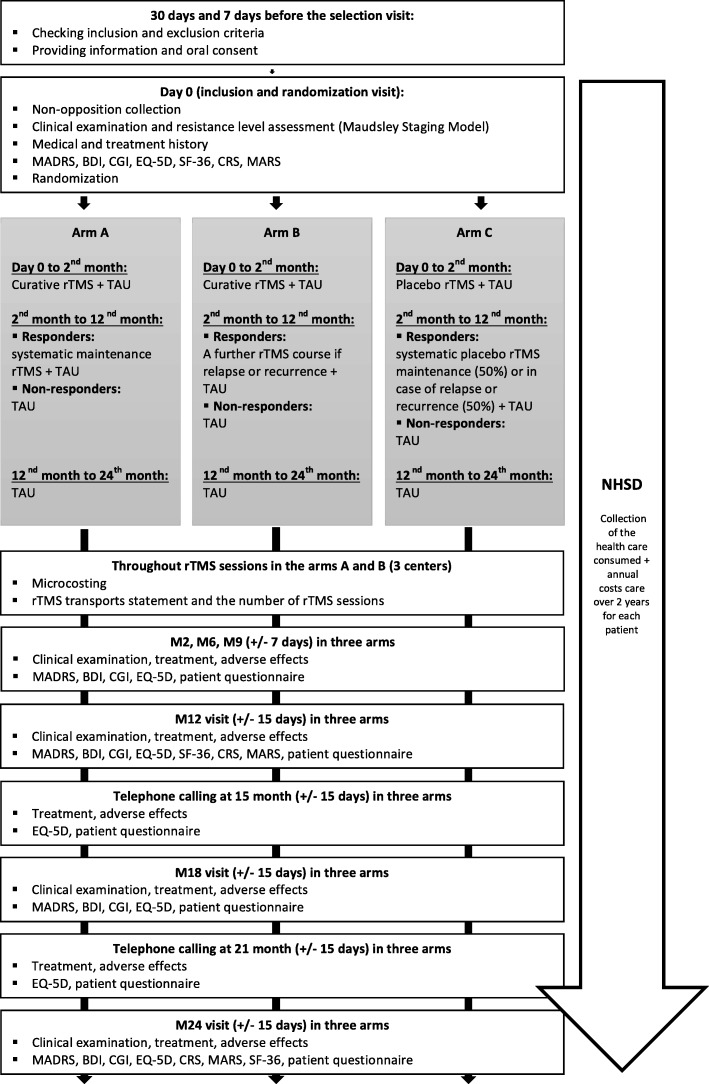
Flow chart

This is a health-economic, prospective, randomized, double-blind, multicenter, parallel-arm study funded by the French Ministry of Health (Reference: 030/2018). The sponsor is the Centre Hospitalier Universitaire (University Hospital) of Nantes, France. The patients are randomly assigned to (Fig. 1):

- ▪ Arm A with active rTMS cure and systematic maintenance M-rTMS for responders (> 50% decrease in MADRS total score);
- ▪ Arm B with a new course of active rTMS at the first sign of depression relapse or recurrence for responders;
- ▪ Arm C with sham cure. In this arm, 50% of responders have systematic placebo rTMS maintenance after the cure (arm C1) and the other 50% have placebo rTMS cure at the first sign of depressive relapse or recurrence (arm C2).

The other standard cares will be conducted as usual in accordance with clinical practices, guidelines, psychiatrists, and patient preferences throughout the study. In the three arms, the non-responders will receive TAU including pharmacotherapy, psychotherapy, and ECT if needed. Moreover, the evaluation of healthcare resources consumed by patients are investigated during the follow-up. The main endpoint is at 12 months, and 24 months for secondary outcome measures. After the 12-month follow-up, the center is free to choose medical care as they wish according to their usual practices (e.g. TAU or rTMS course as needed).

The present study has been approved by the Ethics Committee (reference: 030/2018, RCB no. 2018-A00473–52, last amendment version 2.0 accepted 29 June 2019) and compiled in accordance with the principles of the Declaration of Helsinki (final version 2004) as well as French legislation (article L1121–160 and L1126–7 of the Public Health Code). Participants give their oral consent to take part in the study. Written and verbal information about the study aim and procedures is provided to all the volunteer participants. Investigating physicians will obtain informed consent or assent from potential trial participants. This consent will be traceable in the medical record as recommended by the ethical committee and the French legislation for interventional studies with minimal risk. Information form and consent modalities are available from the corresponding author on request. Participants are asked if they agree to the use of their data should they choose to withdraw from the trial. Participants will also be asked for permission for the research team to share relevant data with people from the University taking part in the research or from regulatory authorities, where relevant. The aim is to enroll 318 patients who have been put forward either by private or hospital psychiatrists, previously informed in writing of the study. There is no anticipated harm or compensation for trial and post-trial participation; this trial does not involve collecting biological specimens for storage.

### Inclusion criteria

The 318 patients aged ≥18 years must present with a current depressive episode with duration between 12 weeks and 3 years considered major according the Diagnostic and Statistical Manual of Mental Disorders, 5th edition (DSM-5) [34], with a MADRS score of ≥ 20 [35] and treatment-resistant with an absence of remission after at least two different antidepressants lines at an effective dosage over a period of 6 weeks during the current episode. TRD includes resistance to antidepressants and validated potentialization agents as quetiapine or lithium [36]. The current treatment may have to be stable for 6 weeks before the baseline and to be continued at a stable dose throughout initial rTMS course. Each individual must be able to: understand the information; take a decision; volunteer to participate; complete the required questionnaire; take orally administrated treatment independently or have the adequate assistance to do so throughout the study; and go to the research center for successive follow-up visits.

### Non-inclusion criteria

Patients presenting with at least one of the following criteria are not enrolled in the study: diagnostic of a depression with psychotic symptoms; current depressive episode better explained by an organic affection or a pharmacological treatment (corticosteroids, interferon, etc.); schizophrenia or psychotic disorder; bipolar disorder; neurologic disease including epilepsy, neurosurgical affection, and significant neurodegenerative disease; intellectual deficiency; a failure of rTMS cure during the current depressive episode, or a personal history of rTMS cure failure, with a protocol using 1 Hz rTMS on the right DLFPC with a minimum of 20 sessions per cure; a failure of ECT cure during the current depressive episode, or a personal history of ECT failure, including a minimum of 12 sessions per cure; history of previous maintenance ECT or at least two course of ECT; contraindication to the practice of rTMS (history of epilepsy or unexplained seizures, neurologic condition such as stroke, trauma, infection, tumor, severe migraine, any ferromagnetic material or implanted device using physiological signals); minors or persons deprived of liberty following a legal or administrative decision or hospitalized without consent, in guardianship; and pregnant and nursing women or women of child-bearing ge who are not using contraception. The same applies for individuals unable to agree with longitudinal follow-up.

### Study process

The screening visit V1 includes the Patient Information Leaflet, collection of the Consent Form and checking of inclusion and exclusion criteria. The medical research teams will be in charge of enrollment and assignation of participants to the intervention. A clinical examination is also carried out and a magnetic resonance imaging (MRI) scan performed in case of neuronavigation use.

### Randomization

During the baseline inclusion visit, the inclusion and exclusion criteria are checked and participants randomized by center into three groups by a computerized random number generator with a permuted block design without stratification or minimization. Participants are randomized to one of the treatment arm using a computer program included in the electronic case report form (eCRF). Initial randomization is performed according the ratio 1:1:0.5:0.5, respectively, corresponding to arm A, arm B, arm C1 (systemic sham rTMS maintenance for responders), and arm C2 (sham rTMS maintenance at the first sign of depressive relapse or recurrence for responders). Block size and type of variation (fixed or randomly) are not known by the investigators to maintain adequate blinding. Randomization occurs in a recorded delay of maximum 15 days before first rTMS or placebo session.

### Blinding

Masking is triple and includes participants, outcome assessors, and investigators. The sham stimulation applied with an identical rTMS procedure at the same location using a commercial figure-eight sham coil. However, it does not produce the same tactile sensation. A local electrical stimulation is delivered with two electromyogram electrodes using a transcutaneous electrical nerve stimulation (TENS) stimulator. At 1 year, patients will be asked to know which treatment (active or placebo) they think they had to evaluate the placebo quality. Just after the randomization, an automatic email with the allocated sequence will be sent to the research nurses who perform the rTMS session in their individual professional mailbox. Investigator and clinical research assessors will be informed of the rTMS schedule (systematical M-TMS or novel rTMS cure) but not the active or sham nature of the treatment.

### Follow-up

**Fig. 2:**
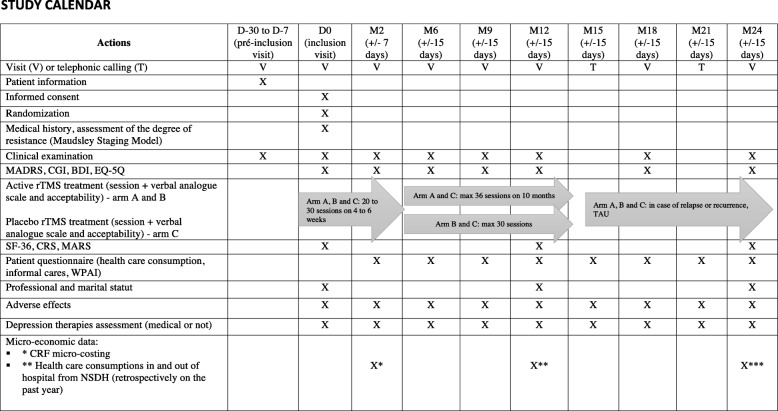
Study calendar

Investigators will meet volunteer participants 2, 6, 9, 12, 18, and 24 months after the last rTMS session (Fig. 2). A patient who fails to respond to therapy (no decrease of > 50% in the MADRS score) despite 6 weeks of treatment will continue longitudinal follow-up to avoid selection bias. A telephone call is realized at 15 and 21 months outside the follow-up consultations to keep in contact with the participants and evaluate the EQ-5D, the patient questionnaire including the Word Productivity and Activity Impairment (WPAI) and formal / informal cares consumption, rTMS side effects, and treatment modifications. The study will last 4 years in total (2018–2022) with 24 months dedicated to the enrollment period and 24 months for longitudinal follow-up (Fig. 1).

### Assessments

The following variables are documented during the baseline visit: sociodemographic (date of birth, age, gender, laterality, professional and marital status); medical history (length of illness, duration of the current episode, co-morbidities (psychiatric, addictive, somatic); and current treatment degree of prior therapeutic resistance according to the Maudsley Staging Model (MSM) [37]).

The following variables are regularly evaluated from the beginning to the end of the study, as shown in Table 1: intensity of the depression according to the MADRS [35]; the Beck Depression Inventory 13 items (BDI 13) [38]; the Clinical Global Impression - Severity (CGI-S) [39]; QALY evaluation by the Euroqol Five Dimensions Questionnaire (EQ-5D) [40]; the quality of life with the Short-Form 36 Health Survey (SF-36) [41]; the drug compliance according the Medication Adherence Rating Scale (MARS) [42] and the Compliance Rating Scale (CRS) [43, 44]; and pharmacological and non-pharmacological treatments statement with the eCRF. The response will be defined by a 50% reduction of MADRS, and remission by MADRS score of < 8. All significant side effects or adverse events will be reported in the eCRF by the research team.

**Table 1:** Hospital production costs in France for a rTMS session

Medical	
Medical time	€39 (US$43.26)
Nurse time	€32 (US$35.50)
Equipment	€33.57 (US$37.24)
Structural expenses	€23.11 (US$25.64)
Total	€127.68 (US$141.64)

Potential explanatory variables to evaluate the depression economic cost are: formal and informal cares consumption (eCRF questionnaire); depression’s socio-professional aftermath with the Work Productivity and Activity Impairment (WPAI) [45] and eCRF questionnaire; health economic analysis with micro-costing; and data extracted from the National Health System Database of Health (NHSD) with the French hospital expenditure database (E.U. Program of Medicalization of Information Systems [PMSI]). Data from the NHSD include: medical consultations; hospitalizations; pharmacological treatments; work stoppages; daily allowances; care-related transports; imagery and biology; nursing cares; and losses of production in association with the WPAI.

### Interventions

Multiple rTMS devices are available in France: Magpro R30 or X100; Magstim Neurostar; Tamas; Powermag; or NBT. All of them feature the Community European (CE) labeling. The most common rTMS devices used are the Magpro R30 or X100 from Magventure (Dantec Company, Copenhagen, Denmark) with an eight-shaped coil and the Magstim Neurostar. The stimulation target is the right dorsolateral prefrontal cortex (Brodmann areas 9 and 46), localized via neuronavigation or by the Beam method. The Beam method, based on the EEG 10/20 target F3, takes into account individual variations and constitutes a valuable approximation of neuronavigation, which is the gold standard [46, 47]. rTMS cures in arms A and B are applied with the following settings: 1 Hz over 8.5 min, 5 days per week (Monday to Friday), 20–30 sessions within 4–6 weeks until remission. The same program is applied in arm C with a sham coil. For systematic rTMS maintenance in arm A, the same parameters are performed with the following rhythm: two sessions per week for 1 month; one session per week for 2 months; and one session every 15 days (36 sessions in total). After the 12-month follow-up, the center is free to choose any therapeutic strategy including M-rTMS or novel rTMS course if needed.

### Primary outcome measures

The primary endpoint in the present study is the ICER analysis (ratio costs / QALY) of these two rTMS therapeutic strategies (arm A or arm B) versus usual chemotherapy, psychotherapy, or ECT without active rTMS (arm C) at 12 months to treat unipolar TRD, according the collective perspective. QALY evaluation is measured by the Euroqol Five Dimension Questionnaire (EQ-5D).

### Secondary outcome measures

The secondary endpoints in each of the three arms are: ICER at 24 months after the last rTMS or placebo session; the cost–utility ratio analysis over 12 and 24 months; 5-year budget impact analysis of the most efficient therapeutic strategies; and prognosis factors of maintenance after a response to an initial rTMS cure. Moreover, the following criteria will be compared between arms A and C, and between arms B and C: rates of responders; remission; disease-free survival at 12 and 24 months; depressive symptomatology evolution; tolerance; observance; treatment modifications; number of suicide attempts and suicide achieved; marital / professional status; and quality of life at 12 and 24 months.

### Statistical analysis

Descriptive analysis of the data collected during each patient evaluation will be carried up until the final evaluation. Specific time points for analysis in the three groups are: baseline; at 1 month after the last rTMS or sham sessions; and 12 and 24 months after the end of the first rTMS or sham course. Continuous variables will be described using median, standard deviation, minimal, maximal, and range for quantitative variables; qualitative variables will be described using frequencies and percentages. No data about quality of life, healthcare consumed, and the cost are available in the literature for this indication, making a priori the calculation of number of individuals needed impossible. The number of participants required for the present study was calculated according to the expected and sustained response in each arm (> 50% decrease in MADRS total score). This rate of responder after the rTMS cure is estimated at 60% in arms A and B at 1 year with 50% of success of rTMS [23, 48]. According to the literature, we hypothesized a rate of 30% maintained response at 1 year for arm A [23, 31, 49, 50] and arm B [23, 50–52], and 11% for arm C [53]. Assuming a 5% (bilateral) type I error, a power of 80%, and an attrition rate of 80%, a total of 318 individuals is required (106 in each arm). The study includes three arms, but only two comparisons will be carried out: arm A versus arm C, and arm B versus arm C.

For the ICER, resources consumed, including costs and QALY, will be presented with average and dispersion for each group. The comparison between cost, QALY, and the years of life gained in each arm, including ICER analysis, will be evaluated with the estimation of their standard deviation and their corresponding 95% confidence intervals (CI) following the non-parametric bootstrap method [54]. Tables will present differences between the three groups including their costs, QALY, and ICER. The ICER is calculated to consider the possibilities of vast dominance between these strategies [55]. For example, the ICER between arms A and C will be calculated as follows:

$$ ICER=\frac{C_{armA}-{C}_{armC}}{QALYs_{armA}-{QALYs}_{armC}} $$

*C*_*armA*_ and *C*_*armC*_ correspond to the costs of depression in each arm and are the QALYs’ gain associated with the rTMS maintenance versus usual cares without rTMS (placebo). At the end, a univariate and determinate sensibility analysis will be estimate with the Tornado method to evaluate the robustness of the resultants concerning the variation in the cost of rTMS sessions and the hypothesis of their pricing.

To estimate the costs of depression, clinical data matching the eCRF and the NHSD will be applied using the patient identification number. If there is a baseline deviation between arms of the study about the quality of life estimated with the EQ-5D, two analyses will be conducted: one with the quality of life raw score; and another with adjusted scores using a logistic regression model [56]. The cost–utility and EQ-5D analysis will be conducted according to the intent-to-treat (ITT) principle on two samples: one sample with completed cases including participants with completed data; and another with an imputed sample in which missing data about cost and quality of life will be imputed according a multiple imputation method.

The rate of remission with their corresponding 95% CIs at 12 and 24 months will be compared between arm A versus arm C, and arm B versus arm C according to the generalized linear model (logistic model) to consider the repeated measurements as a random factor (stratification factor of the randomization). Plots of relapse-free survival at 12 and 24 months will be estimated according to the Kaplan–Meier method; they will be compared between arms A and B, and between arms B and C using the Log-Rank stratified test in each center. A Cox multivariate analysis will be applied to evaluate the sustainability of the response in the time.

MADRS, MARS, CRS, and CGI scores with their corresponding 95% CIs will be compared between arms A and B, and between arms B and C according to a mixed logistic regression model to consider the center and repeated data as random factors. Modifications and side effects of pharmacological treatments will be described and compared using a Chi-squared test (or Fisher’s exact test, if appropriate). The number and percentage of suicidal attempts will be compared in the three arms using Fisher’s exact test. Marital status, professional status, and non-observance percentage (Maudsley Staging Model) will be described in the three arms at 12 and 24 months. The following criteria will be estimated for patients in each arm: responder and remission rate after rTMS or placebo cure; the number of cures prescribed; the duration of the rTMS cures and their side effects; observance rate; and the self-rated pain intensity of the procedure on a verbal analogue scale.

TMS indication is not yet approved in France. To evaluate its cost in the study in the French context, we used the cost-estimate of the rTMS cure calculated in France in 2015 [57]. According to this calculation, the hospital production cost of a rTMS session was estimated at 127.68 Euros (approximately US$141.64 calculated on Thursday, 22 August 2019, with 1 Euro = US$1.11) by session as detailed in Table 1.

## Discussion

The study by Dunner et al. [53] evaluated the effects of TAU among 124 patients affected by a treatment-resistant, non-psychotic major depressive disorder or bipolar depression. They showed a response rate of 11.6% at 12 months and 18.4% at 24 months, with a 3.6% remission rate at 12 months and 7.8% at 24 months, and a poor quality of life globally. The Star*D study among 3671 participants retrieved results along these lines [7]. Indeed, only 20% were responders to an augmentation treatment after the failure of two treatments and 35% of them kept a 1-year profit. After a third treatment line, 28.5% were responders (59% of them were in remission). However, the recurrence rate at 1 year for remitted patients was 43%, and 76% for responders without remission. Pharmacological approaches actually rely on: posology optimization; switch (if inefficiency or intolerance); antidepressant association or potentiation with non-antidepressant treatments; and more or less adjuvant symptomatologic treatments [36, 58]. In addition to these strategies, rTMS may have the potential to improve treatment-resistant unipolar depression [59], with the advantages of being painless, well-tolerated, and twice as effective as placebo [60], even among elderly persons [61]. In a meta-analysis about rTMS efficacy, Kedzior et al. [51] demonstrated a significant decrease of depression scores 16 months after rTMS sessions, with an initial gain over 3 months after the last rTMS cure session. Furthermore, after the failure of one antidepressant treatment, rTMS would be less expensive and would allow a better quality of life and greater function compared to conventional treatment strategies [62]. Although the rTMS is less effective than ECT [32], it is better tolerated and does not require general anesthesia with curarization [48, 63], with a compliance rate of 97% versus approximately 50% for medication. Its mechanisms of action are manifold: improvement in prefrontal metabolism [64], neuromodulation of remote cerebral areas and regulation of the hypothalamo-hypophyseal axis [65]; modulation of cortical excitability [66, 67]; and synaptic plasticity and dopaminergic secretion [68, 69]. However, long-lasting efficacy rTMS is limited to approximately 3–6 months after the curative therapy [70]. It is important to note that a patient who respond to an initial rTMS cure has an 80% chance of responding to another rTMS cure [29], hence the need to evaluate this impact on the prognostic and economic modification.

To be in naturalistic conditions, baseline pharmacological treatments are maintained during the study, especially given the fact that adjuvant rTMS has the potential to increase the efficacy of antidepressant treatments [71]. It is now time to investigate the efficiency and health economic characteristics of these different therapeutic strategies in TRD, including add-on curative and maintenance rTMS versus TAU. To our knowledge, it is the first large prospective trial assessing long-term TMS cost–utility. In our study, the costs of these two strategies will be compared in three steps: collection of resources consumed; unitary cash value of each resource; and the total cost estimation of these strategies realized by multiplying resources consumed by their unitary cash value. It is important to take into account direct costs—with eCRF micro-costing and data extracted from the NHSD over a population matched to the population of the study—and indirect costs of depression. Indeed, the estimation of direct medical and technic costs are insufficient to evaluate the global expenditure in depression. In the present study, indirect costs are estimated by the loss of production during work stoppage and the WPAI assessment. The loss of production is valued by the human capital method. Caregivers’ involvement will also be collected in the eCRF questionnaires to measure the informal costs of depression. According to Knapp et al. [72], measuring the informal costs of depression consists of collecting the number of persons who benefit from different forms of informal care (patient care, childcare, monitoring, domestic assistance) and the time needed to do each of these tasks (in hours per week). Caregiving involvement will be valued at the national hourly charge used for domestic assistance. All these expenditures should be evaluated to estimate the global costs of TRD.

According to health economic criteria, several parameters are necessary to calculate the numbers of individuals in a health economic study including [73]: cost and QALY average deviation over time; costs and QALY standard deviation in each arm; correlation between the costs and QALY difference; and the QALY value. These data are ideally obtained from the literature or a pilot study. However, no source data were available for our study concerning the costs and the quality of life in depression. Moreover, there is no official recommendation available on the QALY monetary value in France [56]. That is why we preferred to calculate the number of individuals required according a clinical criterion, the rate of response at 12 months, rather than a risky health economic hypothesis. This rate of response after the rTMS cure is estimated at 60% in arms A and B, based on the same rTMS protocol already used in French context in that population [48]. According to the literature, we adopted a hypothesis of 30% of sustained response at 1 year in arm A [23, 31, 49, 50] and in arm B [23, 49, 51, 52], and n hypothesis of 11% rate of response sustained at 1 year in arm C [53]. Assuming a 5% (bilateral) type I error, a power of 80%, and an attrition rate of 80%, a total of 318 individuals is required, with 106 in each arm.

With the collection and analysis of all these elements extracted from a large sample with a long-term follow-up, we hope to be able to evaluate the global depression budgetary impact, including TAU and rTMS care, and to highlight the potential economic profit of the most efficient strategy over time after a complete health economic assessment, including data about TAU. Moreover, even today, evidence of utility and the optimal strategy of M-rTMS are still unclear and need more investigation on the long-term use of randomized controlled trials as our study [74]. The present study should provide a data collection near the current clinical practice that will be useful to precise further medico-economic hypothesis in the field. We suppose that add-on rTMS would be more efficient than TAU alone with a better quality of life, a lower rate of hospitalization, a lower rate of medication, and a lower rate of depression in the socio-professional aftermath, including loss of production and work stoppage. One limitation is the use of a single rTMS protocol and the rapid evolution of the field in terms of parameters optimization. Within the study design period we initially chose a low-frequency rTMS protocol to be in line with a previous large study in French context [23], using a short duration protocol that may be of interest for medico-economical purposes, and with less constraints according to the French legislation. Mutz et al. recent meta-analysis supported a good effect size with low-frequency rTMS, as high as other protocols [75], but it did not meet to date a level A of evidence in the rTMS guidelines update [16]. In addition, new shorter rTMS protocols suh as intermittent theta-burst stimulation (iTBS) meanwhile reached high level of evidence and become thus good candidates from a cost-utility perspective [76]. This study aims however at providing comprehensive medico-economical data associated with TRD therapeutic strategies including medications, rTMS, psychotherapy and ECT, and thus may allow simulations with different rTMS protocols (according to response rates, treatment duration and cost), to improve our vision of how to reduce TRD burden and improve long term quality of life.

## Trial status

Patient recruitment starts in November 2018.

## Data Availability

Not applicable.

## Abbreviations

BDI 13: Beck Depression Inventory 13 items
CGI-S: Clinical Global Impression – Severity
CE: Community European
CRS: Compliance Rating Scale
DLPFC: Dorsolateral pre-frontal cortex
DSM-5: Diagnostic and Statistical Manual of Mental Disorders, fifth edition
eCRF: Electronic case report form
ECT: Electroconvulsive therapy
EQ-5D: Euroqol Five Dimension Questionnaire
EEG: Electroencephalogram
EU: European Union
FDA: Food and Drug Administration
ICER: Incremental cost-effectiveness ratio
ITT: Intent-to-treat
LYG: Life years gains
MADRS: Montgomery and Asberg Rating Scale
MARS: Adherence Rating Scale
MRI: Magnetic resonance imaging
M-rTMS: Maintenance repetitive transcranial magnetic stimulation
MSM: Maudsley Staging Model
NHSD: National Health System Database of Health
PMSI: Program of Medicalization of Information Systems
QALY: Quality-adjusted life-years
rTMS: Repetitive transcranial magnetic stimulation
SF-36: Short-Form 36 Health Survey
TAU: Treatment as usual
TENS: Transcutaneous electrical nerve stimulation
TRD: Treatment-resistant depression
WPAI: Word productivity and activity impairment

## References

[1] Ferrari AJ, Charlson FJ, Norman RE, Patten SB, Freedman G, Murray CJ, Vos T, Whiteford HA (2013). Burden of depressive disorders by country, sex, age, and year: findings from the global burden of disease study 2010. PLoS Med.

[2] Lepine JP, Gasquet I, Kovess V, Arbabzadeh-Bouchez S, Negre-Pages L, Nachbaur G (2005). Prevalence and comorbidity of psychiatric disorders in the French general population. L’Encephale.

[3] Gustavsson A, Svensson M, Jacobi F, Allgulander C, Alonso J, Beghi E (2011). Cost of disorders of the brain in Europe 2010. Eur Neuropsychopharmacol.

[4] Stubbs B, Vancampfort D, Veronese N, Kahl KG, Mitchell AJ, Lin P-Y (2017). Depression and physical health multimorbidity: primary data and country-wide meta-analysis of population data from 190 593 people across 43 low-and middle-income countries. Psychol Med.

[5] Pampallona S, Bollini P, Tibaldi G, Kupelnick B, Munizza C (2002). Patient adherence in the treatment of depression. Br J Psychiatry J Ment Sci.

[6] Cleare A, Pariante CM, Young AH, Anderson IM, Christmas D, Cowen PJ (2015). Evidence-based guidelines for treating depressive disorders with antidepressants: a revision of the 2008 British Association for Psychopharmacology guidelines. J Psychopharmacol (Oxf).

[7] Rush AJ, Trivedi MH, Wisniewski SR, Nierenberg AA, Stewart JW, Warden D (2006). Acute and longer-term outcomes in depressed outpatients requiring one or several treatment steps: a STAR* D report. Am J Psychiatry.

[8] Fava M (2003). Diagnosis and definition of treatment-resistant depression. Biol Psychiatry.

[9] Opening_Eyes_Report_En_2012.pfd. https://www.publichealthontario.ca/fr/eRepository/Opening_Eyes_Report_En_2012.pdf. Accessed 11 Sept 2017.

[10] Trivedi MH, Rush AJ, Wisniewski SR, Nierenberg AA, Warden D, Ritz L (2006). Evaluation of outcomes with citalopram for depression using measurement-based care in STAR* D: implications for clinical practice. Am J Psychiatry.

[11] Fawcett J (1994). Overview of mood disorders: diagnosis, classification, and management. Clin Chem.

[12] Miller IW, Keitner GI, Schatzberg AF, Klein DN, Thase ME, Rush AJ (1998). The treatment of chronic depression, part 3: psychosocial functioning before and after treatment with sertraline or imipramine. J Clin Psychiatry.

[13] Gibson TB, Jing Y, Smith Carls G, Kim E, Bagalman JE, Burton WN (2010). Cost burden of treatment resistance in patients with depression. Am J Manag Care.

[14] DiMatteo MR, Lepper HS, Croghan TW (2000). Depression is a risk factor for noncompliance with medical treatment: meta-analysis of the effects of anxiety and depression on patient adherence. Arch Intern Med.

[15] Heijnen WT, Birkenhäger TK, Wierdsma AI, van den Broek WW (2010). Antidepressant pharmacotherapy failure and response to subsequent electroconvulsive therapy: a meta-analysis. J Clin Psychopharmacol.

[16] Lefaucheur JP, Aleman A, Baeken C, Benninger DH, Brunelin J, Di Lazzaro V, et al. Evidence-based guidelines on the therapeutic use of repetitive transcranial magnetic stimulation (rTMS): An update (2014-2018). Clin Neurophysiol. 2020;131(2):474-528. 10.1016/j.clinph.2019.11.002 31901449

[17] Milev RV, Giacobbe P, Kennedy SH, Blumberger DM, Daskalakis ZJ, Downar J (2016). Canadian Network for Mood and Anxiety Treatments (CANMAT) 2016 Clinical Guidelines for the Management of Adults with Major Depressive Disorder: Section 4. Neurostimulation Treatments. Can J Psychiatry Rev Can Psychiatr.

[18] Wang Y-M, Li N, Yang L-L, Song M, Shi L, Chen W-H (2017). Randomized controlled trial of repetitive transcranial magnetic stimulation combined with paroxetine for the treatment of patients with first-episode major depressive disorder. Psychiatry Res.

[19] Rumi DO, Gattaz WF, Rigonatti SP, Rosa MA, Fregni F, Rosa MO (2005). Transcranial magnetic stimulation accelerates the antidepressant effect of amitriptyline in severe depression: a double-blind placebo-controlled study. Biol Psychiatry.

[20] Chen J, Zhou C, Wu B, Wang Y, Li Q, Wei Y (2013). Left versus right repetitive transcranial magnetic stimulation in treating major depression: a meta-analysis of randomised controlled trials. Psychiatry Res.

[21] Berlim MT, Van den Eynde F, Jeff DZ (2013). Clinically meaningful efficacy and acceptability of low-frequency repetitive transcranial magnetic stimulation (rTMS) for treating primary major depression: a meta-analysis of randomized, double-blind and sham-controlled trials. Neuropsychopharmacol Off Publ Am Coll Neuropsychopharmacol.

[22] O’Reardon JP, Solvason HB, Janicak PG, Sampson S, Isenberg KE, Nahas Z (2007). Efficacy and safety of transcranial magnetic stimulation in the acute treatment of major depression: a multisite randomized controlled trial. Biol Psychiatry.

[23] Brunelin J, Jalenques I, Trojak B, Attal J, Szekely D, Gay A (2014). The efficacy and safety of low frequency repetitive transcranial magnetic stimulation for treatment-resistant depression: the results from a large multicenter French RCT. Brain Stimul.

[24] Nadeau SE, Bowers D, Jones TL, Wu SS, Triggs WJ, Heilman KM (2014). Cognitive effects of treatment of depression with repetitive transcranial magnetic stimulation. Cogn Behav Neurol Off J Soc Behav Cogn Neurol.

[25] Valiengo LCL, Benseñor IM, Lotufo PA, Fraguas R, Brunoni AR (2013). Transcranial direct current stimulation and repetitive transcranial magnetic stimulation in consultation-liaison psychiatry. Braz J Med Biol Res Rev Bras Pesqui Medicas E Biol.

[26] Solvason HB, Husain M, Fitzgerald PB, Rosenquist P, McCall WV, Kimball J (2014). Improvement in quality of life with left prefrontal transcranial magnetic stimulation in patients with pharmacoresistant major depression: acute and six month outcomes. Brain Stimulat.

[27] Rosenberg O, Dinur Klein L, Gersner R, Kotler M, Zangen A, Dannon P (2015). Long-term Follow-up of MDD Patients Who Respond to Deep rTMS: A Brief Report. Isr J Psychiatry Relat Sci.

[28] Kellner CH, Knapp RG, Petrides G, Rummans TA, Husain MM, Rasmussen K (2006). Continuation electroconvulsive therapy vs pharmacotherapy for relapse prevention in major depression: a multisite study from the Consortium for Research in Electroconvulsive Therapy (CORE). Arch Gen Psychiatry.

[29] Kelly MS, Oliveira-Maia AJ, Bernstein M, Stern AP, Press DZ, Pascual-Leone A (2017). Initial Response to Transcranial Magnetic Stimulation Treatment for Depression Predicts Subsequent Response. J Neuropsychiatry Clin Neurosci.

[30] Janicak PG, Nahas Z, Lisanby SH, Solvason HB, Sampson SM, McDonald WM (2010). Durability of clinical benefit with transcranial magnetic stimulation (TMS) in the treatment of pharmacoresistant major depression: assessment of relapse during a 6-month, multisite, open-label study. Brain Stimulat.

[31] Connolly KR, Helmer A, Cristancho MA, Cristancho P, O’Reardon JP (2012). Effectiveness of transcranial magnetic stimulation in clinical practice post-FDA approval in the United States: results observed with the first 100 consecutive cases of depression at an academic medical center. J Clin Psychiatry.

[32] Health Quality Ontario (2016). Repetitive Transcranial Magnetic Stimulation for Treatment-Resistant Depression: An Economic Analysis. Ont Health Technol Assess Ser.

[33] Nguyen K-H, Gordon LG (2015). Cost-Effectiveness of Repetitive Transcranial Magnetic Stimulation versus Antidepressant Therapy for Treatment-Resistant Depression. Value Health J Int Soc Pharmacoeconomics Outcomes Res.

[34] American Psychiatric Association (2013). DSM-5: diagnostic and statistical manual of mental disorders.

[35] Montgomery SA, Asberg M (1979). A new depression scale designed to be sensitive to change. Br J Psychiatry J Ment Sci.

[36] Charpeaud T, Genty JB, Destouches S, Yrondi A, Lancrenon S, Alaïli N (2017). French Society for Biological Psychiatry and Neuropsychopharmacology and Fondation FondaMental task force: Formal Consensus for the management of treatment-resistant depression. Encephale.

[37] Fekadu A, Wooderson S, Donaldson C, Markopoulou K, Masterson B, Poon L (2009). A multidimensional tool to quantify treatment resistance in depression: the Maudsley staging method. J Clin Psychiatry.

[38] Beck AT, Beamesderfer A (1974). Assessment of depression: the depression inventory. Mod Probl Pharmacopsychiatry.

[39] Guy W (1976). ECDEU assessment manual for psychopharmacology.

[40] Rabin R, de Charro F (2001). EQ-SD: a measure of health status from the EuroQol Group. Ann Med.

[41] McHorney CA, Ware JE, Lu JF, Sherbourne CD (1994). The MOS 36-item Short-Form Health Survey (SF-36): III. Tests of data quality, scaling assumptions, and reliability across diverse patient groups. Med Care.

[42] Thompson K, Kulkarni J, Sergejew AA (2000). Reliability and validity of a new Medication Adherence Rating Scale (MARS) for the psychoses. Schizophr Res.

[43] Hogan TP, Awad AG, Eastwood R (1983). A self-report scale predictive of drug compliance in schizophrenics: reliability and discriminative validity. Psychol Med.

[44] Kemp R, Kirov G, Everitt B, Hayward P, David A (1998). Randomised controlled trial of compliance therapy. 18-month follow-up. Br J Psychiatry J Ment Sci.

[45] Reilly MC, Zbrozek AS, Dukes EM (1993). The validity and reproducibility of a work productivity and activity impairment instrument. Pharmacoeconomics.

[46] Beuzon G, Timour Q, Saoud M (2017). Predictors of response to repetitive transcranial magnetic stimulation (rTMS) in the treatment of major depressive disorder. L’Encephale.

[47] Mir-Moghtadaei A, Caballero R, Fried P, Fox MD, Lee K, Giacobbe P (2015). Concordance Between BeamF3 and MRI-neuronavigated Target Sites for Repetitive Transcranial Magnetic Stimulation of the Left Dorsolateral Prefrontal Cortex. Brain Stimulat.

[48] Brunelin J, Ben Maklouf W, Nicolas A, Saoud M, Poulet E (2010). Successful switch to maintenance rTMS after maintenance ECT in refractory bipolar disorder. Brain Stimulat.

[49] Richieri R, Guedj E, Michel P, Loundou A, Auquier P, Lançon C (2013). Maintenance transcranial magnetic stimulation reduces depression relapse: a propensity-adjusted analysis. J Affect Disord.

[50] Haesebaert F, Moirand R, Schott-Pethelaz A-M, Brunelin J, Poulet E (2018). Usefulness of repetitive transcranial magnetic stimulation as a maintenance treatment in patients with major depression. World J Biol Psychiatry Off J World Fed Soc Biol Psychiatry.

[51] Kedzior KK, Reitz SK, Azorina V, Loo C (2015). Durability of the antidepressant effect of the high-frequency repetitive transcranial magnetic stimulation (rTMS) In the absence of maintenance treatment in major depression: a systematic review and meta-analysis of 16 double-blind, randomized, sham-controlled trials. Depress Anxiety.

[52] Cohen RB, Boggio PS, Fregni F (2009). Risk factors for relapse after remission with repetitive transcranial magnetic stimulation for the treatment of depression. Depress Anxiety.

[53] Dunner DL, Rush AJ, Russell JM, Burke M, Woodard S, Wingard P (2006). Prospective, long-term, multicenter study of the naturalistic outcomes of patients with treatment-resistant depression. J Clin Psychiatry.

[54] Efron B, Tibshirani R (1997). Improvements on cross-validation: the 632+ bootstrap method. J Am Stat Assoc.

[55] Drummond MF, Sculpher MJ, Claxton K, Stoddart GL, Torrance GW (2015). Methods for the economic evaluation of health care programmes.

[56] Collège de la Haute Autorité de Santé. Valeurs de référence pour l’évaluation économique en santé. Haute Autorité de Santé: Service communication –information; 2, avenue du Stade de France –F 93218 Saint-Denis La Plaine; 2014.

[57] Etcheverrigaray F, Bulteau S, Machon LO, Riche VP, Mauduit N, Tricot R (2015). [Hospital production cost of repetitive transcranial magnetic stimulation (rTMS) in the treatment of depression]. Rev Épidémiologie Santé Publique. Rev Épidémiologie Santé Publique.

[58] NICE. Depression in adults: recognition and management / Guidance and guidelines / NICE. https://www.nice.org.uk/guidance/cg90. Accessed 11 Sept 2017.

[59] McGirr A, Berlim MT (2018). Clinical Usefulness of therapeutic neuromodulation for major depression: A systematic meta-review of recent meta-analyses. Psychiatr Clin.

[60] Berlim MT, Van den Eynde F, Daskalakis ZJ (2013). High-frequency repetitive transcranial magnetic stimulation accelerates and enhances the clinical response to antidepressants in major depression: a meta-analysis of randomized, double-blind, and sham-controlled trials. J Clin Psychiatry.

[61] Ciobanu C, Girard M, Marin B, Labrunie A, Malauzat D (2013). rTMS for pharmacoresistant major depression in the clinical setting of a psychiatric hospital: effectiveness and effects of age. J Affect Disord.

[62] Simpson KN, Welch MJ, Kozel FA, Demitrack MA, Nahas Z (2009). Cost-effectiveness of transcranial magnetic stimulation in the treatment of major depression: a health economics analysis. Adv Ther.

[63] Jin X-L, Xu W-Q, Le Y-J, Dai X-K (2016). Long-term Effectiveness of Modified Electroconvulsive Therapy Compared With Repetitive Transcranial Magnetic Stimulation for the Treatment of Recurrent Major Depressive Disorder. J Nerv Ment Dis.

[64] Bestmann S, Baudewig J, Siebner HR, Rothwell JC, Frahm J (2005). BOLD MRI responses to repetitive TMS over human dorsal premotor cortex. Neuroimage.

[65] Baeken C, Marinazzo D, Eveaert H, Wu GR, Van Hove C, Andenaert K (2015). The impact of accelerated HF-rTMS on the subgenual anterior cigulate cortex in refractory unipolar major depression: insights from (18) FDG PET brain imaging. Brain Stimul.

[66] Baeken C, De Raedt R (2011). Neurobiological mechanisms of repetitive transcranial magnetic stimulation on the underlying neurocircuit in unipolar depression. Dialogues Clin Neurosci.

[67] Hoogendam JM, Ramakers GMJ, Di Lazzaro V (2010). Physiology of repetitive transcranial magnetic stimulation of the human brain. Brain Stimul.

[68] Shaul U, Ben-Shachar D, Karry R, Klein E (2003). Modulation of frequency and duration of repetitive magnetic stimulation affects catecholamine levels and tyrosine hydroxylase activity in human neuroblastoma cells: implication for antidepressant effect of rTMS. Int J Neuropsychopharmacol.

[69] Strafella AP, Paus T, Fraraccio M, Dagher A (2003). Striatal dopamine release induced by repetitive transcranial magnetic stimulation of te human motor cortex. Brain.

[70] Lam RW, Chan P, Wilkins-Ho M, Yatham LN (2008). Repetitive transcranial magnetic stimulation for treatment-resistant depression: a systematic review and metaanalysis. Can J Psychiatry Rev Can Psychiatr.

[71] Fitzgerald PB, Grace N, Hoy KE, Bailey M, Daskalakis ZJ (2013). An open label trial of clustered maintenance rTMS for patients with refractory depression. Brain Stimulat.

[72] Knapp M, Romeo R, Mogg A, Eranti S, Pluck G, Purvis R (2008). Cost-effectiveness of transcranial magnetic stimulation vs. electroconvulsive therapy for severe depression: a multi-centre randomised controlled trial. J Affect Disord.

[73] Glick HA (2011). Sample size and power for cost-effectiveness analysis (part 1). Pharmacoeconomics.

[74] Senova S, Cotovio G, Pascual-Leone A, Oliveira-Maia AJ (2019). Durability of antidepressant response to repetitive transcranial magnetic stimulation: Systematic review and meta-analysis. Brain Stimulat.

[75] Mutz J, Vipulananthan V, Carter B, Hurlemann R, Fu CH, Young AH (2019). Comparative efficacy and acceptability of non-surgical brain stimulation for the acute treatment of major depressive episodes in adults: systematic review and network meta-analysis. BMJ.

[76] Blumberger DM, Vila-Rodriguez F, Thorpe KE, Feffer K, Noda Y, Giacobbe P, et al. Effectiveness of theta burst versus high-frequency repetitive transcranial magnetic stimulation in patients with depression (THREE-D): a randomised non-inferiority trial. The Lancet. 2018;391(10131):1683-92. 10.1016/S0140-6736(18)30295-2 29726344

